# *Fat Mass Obesity* Gene Predisposing in Preeclampsia in the Madura Population: A Case-Control Study

**DOI:** 10.34763/jmotherandchild.20263001.d-26-00010

**Published:** 2026-07-18

**Authors:** Eny Susanti, Zakkiyatus Zainiyah, Novita Wulandari, Mochamad Amin, Iswahyudi

**Affiliations:** Department of Midwifery Study Program, Noor Huda Mustofa University, East Java, Indonesia; Institute of Tropical Disease, Universitas Airlangga, Surabaya, 60115, East Java, Indonesia

**Keywords:** FTO, gene, Madura, normotension, preeclampsia

## Abstract

**Background:**

Preeclampsia (PE) is a common complication of pregnancy characterised by hypertension, severe headaches, and protein in the urine, usually developing after 20 weeks of pregnancy. High body mass index before pregnancy is a risk factor for PE. One gene associated with obesity is the Fat Mass Obesity (FTO) gene. This study aimed to analyse the FTO gene predisposing to PE in the Madura population.

**Material and Methods:**

The research design is a case-control design. A total of 110 pregnant women participated: 55 with severe PE and 55 healthy pregnant women. The inclusion criteria for the case group were patients with a history of PE who visited Syamrabu Bangkalan Regional Hospital and the independent midwifery practice, and who were of Madurese ethnicity. Blood samples were collected for FTO gene analysis; a questionnaire was used to obtain demographic data from pregnant women; and the FTO gene was analysed by tetra-primer amplification refractory mutation system polymerase chain reaction method, followed by sequencing and statistical analysis using Statistical Package for the Social Sciences (SPSS).

**Results:**

In normotensive pregnant women, the AA homozygous genotype was less common than in the PE group (1.8% vs 12.7%). The results were statistically significant (p = 0.018) between normotensive and preeclamptic pregnant women.

**Conclusion:**

The FTO gene may be a risk factor for PE and is associated with the AA homozygous genotype; further research with large sample sizes across diverse ethnicities is needed.

## Introduction

Hypertension that occurs during pregnancy is called preeclampsia (PE), which affects 5%–10% of pregnancies, and its prevalence continues to increase [1,2,3]. PE is an idiopathic multisystem condition characterised by hypertension ≥140/90 mmHg, proteinuria ≥300 mg/24 hr after 20 weeks of gestation [4,5]. Severe PE can cause kidney, heart, lung, liver, and neurological dysfunction; haematological disorders; foetal growth disorders, stillbirth; and maternal death [6,7]. Prevention of PE has been widely developed today, starting from early detection of pregnancies <20 weeks to identify risk factors for PE so that appropriate treatment can be provided to pregnant women to prevent severe complications. In addition, medications such as low-dose aspirin for pregnant women at high risk of PE and non-pharmacological prevention, such as light exercise, walking, prenatal yoga, deep breathing, and many other preventive measures have been previously studied. However, the results are still conflicting, so further research is needed [8,9,10]. Cases of PE are increasing year by year, so early detection and appropriate prevention are needed, considering the short- and long-term impacts caused by PE, which will lead to eclampsia or other complications such as maternal and foetal death.

PE is a major complication in pregnant women, affecting both the mother and the foetus, and is closely associated with a high body mass index (BMI) [11]. Risk factors for PE include a history of PE, diabetes, hypertension, obesity, multiple pregnancies, and psychological stress [12]. Genetic and environmental factors interact to significantly influence the risk of PE. Several emerging studies suggest that the predisposition to PE may be due to genetic components associated with hypertension and obesity, such as Angiotensin II receptor type 1 (*ATR1), ACE*, and *FTO*, but previous research findings are conflicting [10]. One of the factors causing PE is obesity, and the *fat mass obesity* (*FTO*) gene is one of the genetic factors associated with obesity and PE.

The occurrence of PE is influenced by many factors, one of which is the *FTO* gene [13]. Previous research has shown that *FTO* gene variants are associated with increased body fat mass and obesity. *FTO* gene expression is found in the brain, liver, and adipose tissue. In addition, the *FTO* gene plays a key role in regulating fat metabolism and disease risk. Studies in mouse models have found that *FTO* hyperactivity increases fat accumulation, while *FTO* inhibition reduces body fat [14,15]. Research in the last decade has shown that variations in the *FTO* gene associated with fat and obesity have received significant attention in the regulation of body weight, adiposity, and insulin resistance [16]. The fat mass and obesity associated *FTO* gene is a risk factor for PE, but findings remain disputed [13]. Excess weight and genetic factors contribute to 40%–70% of the occurrence of a disease, especially degenerative diseases, and obesity is a risk factor for PE [17]. To date, maternal genetic variants that contribute to PE risk remain unknown, despite extensive genetic research. Previous studies conducted in the Iranian population revealed that the *FTO* rs9939609 polymorphism, for the first time, was possibly associated with a low risk of PE under a recessive inheritance pattern [13].

The *FTO* gene was the first obesity susceptibility gene identified through a genome-wide association study (GWAS). A growing body of research indicates that *FTO* genetic variants are strongly associated with the risk of cardiovascular disease, including hypertension and acute coronary syndrome [18]. The human *FTO* gene encodes an RNA demethylase and is expressed in Fe (II)/2-O2, suggesting a role in regulating gene expression by modulating its methylation-demethylation state. Previous research has revealed that the *FTO* gene is found in tissues such as adipose tissue and skeletal muscle. A genetic study identified the *FTO* gene as a locus associated with obesity, and several single-nucleotide polymorphisms (SNPs) in intron 1 were strongly associated with BMI, body fat content, waist and hip circumference, and energy intake. Furthermore, the *FTO* gene is expressed in the hippocampus, cerebellum, and hypothalamus, which regulate energy balance in the body [11]. These SNPs also regulate *FTO* gene expression and function as enzymatic catabolites of the *FTO* protein [19]. Data on the *FTO* gene and PE are very limited, despite several studies showing that obesity is associated with an increased incidence of PE, underscoring the need for further research, particularly in the Madurese population.

National data shows that PE is the second-highest cause of maternal death. In East Java, PE ranks first in maternal death, especially in Bangkalan Regency, Madura. East Java Health Service data in 2022 show that the number of maternal deaths was as many as 499. The most common causes are pregnancy, hypertension disorders/PE (24.45%), and bleeding (21.24%). The highest number of mothers is in Pamekasan Regency (30), Bangkalan (16), and Sampang and Sumenep (12) (East Java Health Profile 2022) [20]. Data on PE/eclampsia cases in 2023 at Syamrabu Bangkalan Regional Hospital are around 60–70 cases per month, while data on PE/eclampsia at the Bangkalan Regency Health Service are around 519 in 2023; the highest number of cases is in Kokop District, with as many as 132. The high incidence of PE cases is in Madura, and until now, the exact cause is unknown. Various risk factors have been associated with an increased risk of PE, and the *FTO* gene is of particular interest. Therefore, it is necessary to study the *FTO* genetic factors associated with obesity, a risk factor for PE, to develop early screening and appropriate prevention strategies to reduce complications. The incidence of PE in the Madurese population is still high, and genetics research, especially in the Madurese population, is still not available. The incidence of PE, until now, is not known for certain. As seen from the characteristics of pregnant women who experience PE, they are obese, based their BMI data. The average BMI is 30.12 kg/m2, based on interviews with pregnant women from rural areas who rarely move or exercise, and who are usually obese before pregnancy. The purpose of this study was to analyse the association between the *FTO* gene and PE in the Madura population.

## Material and methods

### Study design and setting

This is a case-control study design conducted at the Syamrabu Bangkalan Regional Hospital and the independent midwifery practice, or in the community, from 14 June to 15 August 2024.

### Participants

The number of respondents was 110 pregnant women, 55 pregnant women with severe PE, 55 healthy pregnant women, in the case group, according to the inclusion and exclusion criteria. The inclusion criteria for the case group were PE patients who attended the programme and were third generation Madurese natives with either early or onset PE, single, with blood pressure ≥140/90 mmHg, proteinuria ≥0.3 g/24 hr, or ≥+1 from the dip paper. Exclusion criteria applied for eclampsia and Hemolysis, Elevated Liver enzymes and Low Platelets (HELLP) syndrome.

The control group comprised healthy pregnant women (control group criteria: all gestational ages, without PE or chronic hypertension, willing to participate, aged 20–35 years), totalling 55 participants. Samples were collected during pregnancy check-ups at the independent midwifery practice or in the community.

### Variables

The sample size was taken based on the case-control sample formula; sampling was taken purposively according to the inclusion and exclusion criteria, both in hospitals and independent midwifery practices. The case and control groups had previously provided written explanations and informed consent. After that, interviews were conducted to collect demographic data from pregnant women, including age, parity, pre-pregnancy weight, systolic and diastolic blood pressure, and Mean Arterial Pressure (MAP). Other biomarker examinations, such as serum cortisol, Angiotensin-Converting Enzyme (ACE), and (VEGF), were performed using an enzyme-linked immunoassay method (ELISA) Kit at the Institute of Tropical Disease, Airlangga University, Surabaya. They agreed and signed an informed consent agreement.

### DNA isolation and genotyping

Blood samples were collected during visits by pregnant women who underwent an Integrated Antenatal Care examination conducted by a midwife. Pregnant women were interviewed to collect data on their characteristics. Samples that met the inclusion criteria were then continued with peripheral blood mononuclear cell (PBMC) examination from separated blood, then PBMC DNA was extracted and amplified in the *FTO* gene region using the tetra-primer amplification refractory mutation system polymerase chain reaction method (TP-ARMS)-polymerase chain reaction (PCR)) technique, using blood, initial denaturation at 95°C for 5 min, followed by 35 cycles of denaturation at 94°C for 2 min and a second denaturation at 95°C for 30′ with 35 cycles, annealing at 59.5°C for 50′ and extension at 72°C for 45′. The final cycle was carried out with a final extension for 5 min at 72°C. The *FTO* gene was then sequenced without Restriction Fragment Length Polymorphism (RFLP). Four *FTO* gene fragments were obtained using the following nucleotides: Forward outer 5′-AGTTCCAGTCATTTTTGACAGC-3′, Reverse outer 5′-AGCCTCTCTACCATCTTATGTC-3′, Forward inner5′-CCTTGCGACTGCTGTGAATATA-3′, Reverse inner 5′-GAGACTATCCAAGTGCATCTCA-3′, PCR products with 2 alleles of 429 and 194 bp. Examination of ACE, cortisol, and VEGF levels using serum from blood taken through veins from pregnant women using the ELISA kit [21,22].

### Bias

To reduce bias in this study and influence the results of the study, samples were taken from the Madurese population of three generations, and controls were not taken from the Hospital but were taken in the Community. Pregnant women who visited the independent midwifery practice, to reduce bias from controls taken with almost the same characteristics, the examination was carried out on fresh samples to get good DNA results to reduce this bias. Strict ethnic matching and strong laboratory examination control were carried out.

### Ethics

Ethical approval was obtained at the School of Health Science with Ethics No. 2313/KEPK/STIKes-NHM/EC/VI/2024.

### Statistical analysis

Data were analysed by computing means and standard deviations for quantitative variables and by examining frequency distributions for categorical variables. Normality test was conducted using the Kolmogorov-Smirnov test. For demographic data that use ratio data, use the mean (Standard Deviation); for categorical data, use percentages. If the data are normally distributed, use the independent *t*-test; if the distribution is not normal, use the Mann–Whitney test; and for the Chi-Square test, use the *FTO* gene analysis. Hardy–Weinberg equilibrium was observed for the examined SNPs. All data were analysed using IBM SPSS Statistics for Windows, Version 29.0. Based on the 95% confidence interval, odds ratio was calculated and presented. Statistical significance was defined as a *p*-value <0.05.

## Result

The results of the study showed differences between preeclamptic and healthy pregnant women without risk factors for PE. The analysis results were significantly different in age (<0.001), parity (0.024), pre-pregnancy weight (<0.001), BMI (<0.001), systolic (<0.001), diastolic (<0.001), MAP (0.001), and cortisol (0.008) (Table 1).

**Table 1.** Characteristics and results of biomarker examination.

**Characteristic**	**Description/category**	**PE (55)**	**Normotension (55)**	**Signifikan**
Age (years)	Mean ± SD (Min-Maks)	32.86 ± 6.61 (21–44)	25.63 ± 3.10 (21–31)	<0.001^b^
Parity	Primigravida	13.8	17.2	0.024^c^
	Multigravida	34.5	19.0	
	Grandemultigravida	13.8	1.7	
Pre-pregnancy weight	Mean ± SD (Min-Maks)	69.75 ± 18.71 (49.00–125.00)	47.19 ± 6.45 (35.00–59.00)	<0.001^b^
BMI (kg/m^2^)	Mean ± SD (Min-Maks)	30.12 ± 7.04 (18.30–48.80)	20.45 ± 2.31 (15.40–24.60)	<0.001^a^
Sistole (mmHg)	Mean ± SD (Min-Maks)	161.11 ± 17.34 (117–193)	104.54 ± 7.01 (90–122)	<0.001^b^
Diastole (mmHg)	Mean ± SD (Min-Maks)	101.11 ± 14.21 (76–130)	68.45 ± 6.55 (60–86)	<0.001^a^
MAP	Mean ± SD (Min-Maks)	121.05 ± 13.42 (100–150)	80.31 ± 6.31 (70–98)	<0.001^b^
Cortisol	Mean ± SD (Min-Maks)	47.20 ± 37.94 (10.39–162.56)	24.27 ± 14.62 (4.18–49.40)	0.008^b^
ACE	Mean ± SD (Min-Maks)	66.89 ± 49.18 (32.57–216.47)	47.32 ± 16.99 (24.39–100.69)	0.089^b^
VEGF	Mean ± SD (Min-Maks)	699.56 ± 692.87 (185.05–3837.00)	557.18 ± 334.32 (235.53–1404.51)	0.226^b^

ACE, angiotensin-converting enzyme; BMI, body mass index; MAP, mean arterial pressure; PE, preeclampsia; SD; standard deviation; VEGF, vascular endothelial growth factor.

Statistik ^a^T test, ^b^Mann–Whitney, ^c^Rank Spearman.

The study results in preeclamptic pregnant women, compared with those in healthy controls, showed a different genotype distribution. In normotensive women, the *AA homozygous* genotype at rs9939609 was significantly less frequent than in the PE group (1.8% vs 12.7%). The *FTO* gene inheritance model showed AA codominance (*p* = 0.018), *AA*/*AT* + *TT* dominance (*p* = 0.028), and *A* and *T* alleles (*p* = 0.038) as potentially associated with PE (Table 2).

**Table 2.** Analysis gene *FTO* pada PE and normotension

**Model**	**Gene *FTO***	**Normotension (*n* = 55)**	**PE (*n =* 55)**	**Chi2**	***p*-value**	**OR (95% CI)**
Codominant	*AA*	1	7	5.627	0.018*	0.016 (0.012–0.922)
	*AT*	23	25	3.512	0.120	0.155 (0.018–1.361)
	*TT*	31	23	0.919	0.338	0.683 (0.312–1.492)
Dominant	*AA*/*AT* + *TT*	1/54	7/48	4.853	0.028*	0.127 (0.015–1.070)
Recessive	*AA* + *AT*/*TT*	24/31	32/23	2.328	0.127	0.556 (0.261–1.185)
Alel *A*		25	39	4.319	0.038*	0.535 (0.296–0.969)
Alel *T*		85	76			

* *p* < 0.05 (statistically significant).

OR, odds ratios; PE, preeclampsia

Uji statistic Chi-Square.

## Discussion

**Figure 1.**
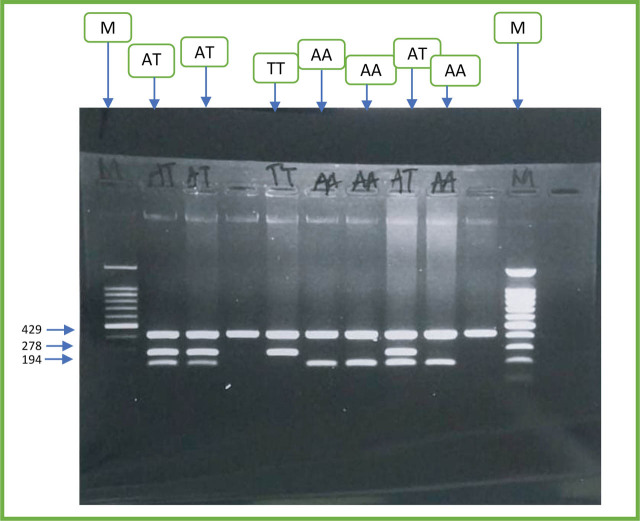
Electrophoresis gene *FTO* rs9939609 genotypes. Marker (M) 1000 bp DNA ladder, 1 Pita NTC, 3 Pita *AT* genotype (uk 194, 278, and 429 bp), 2 Pita *TT* genotype (uk 278 and 429 bp), and 2 Pita *AA* genotype (uk 194 and 429 bp). NTC, non-template control.

Research in the Madurese population found that the *FTO* gene has a codominant, dominant, and allelic inheritance model, potentially associated with PE (Figure 1). The AA genotype of the *FTO* gene is associated with a lower risk of PE. This study investigated the association of the *FTO* gene with PE. The *FTO* gene was identified as the first gene associated with obesity, and several previous studies have shown that *FTO* has an important role in obesity [13]. In line with research in the Madurese population, it was found that normotensive and preeclamptic pregnant women differed significantly in terms of BMI, and the average BMI of preeclamptic pregnant women was obese. This study is consistent with research conducted in Iran, which has found that the *FTO* gene polymorphism rs9939609 is associated with PE under dominant, codominant, and recessive inheritance models [13]. Studies have revealed that the *FTO* gene is located on human chromosome 16q12.2 and consists of nine exons and eight introns. GWAS have shown that the *A* allele of the *FTO* intronic variant rs9939609 increases the risk of obesity in humans [19]. The protein encoded by *FTO* is the *Fat Mass Obesity (FTO)* protein. The *FTO* protein has been shown to play a role in adipogenesis through the mammalian target of rapamycin pathway and post-transcriptional control of downstream gene expression. The association between the *FTO* gene and PE may offer important clues to its genetic basis and provide a basis for early detection [19]. Polygenic Risk Score (PRS) analysis results indicate that a genetic predisposition to obesity influences the risk of PE, a condition largely caused by increased body weight. *FTO* gene variants are most strongly associated with body fat and increased blood pressure [23].

**Figure 2.**
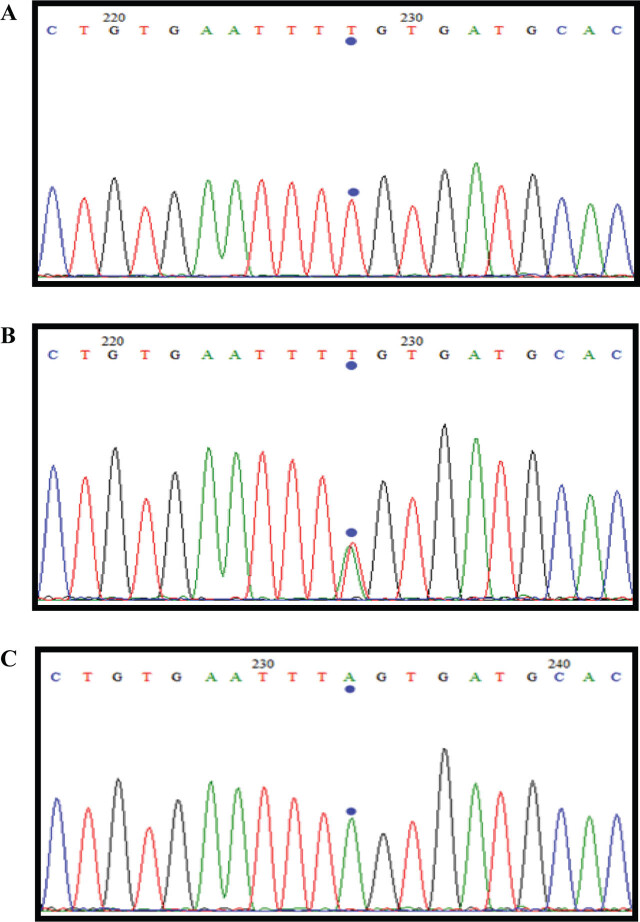
Sequencing gene *FTO* rs9939609 polymorphism. **(A)** *TT* genotype; **(B)** *AT* genotype; **(C)** *AA* genotype.

Evidence from multiple studies suggests an association between the *FTO* gene polymorphism rs9939609 and serum lipid profiles. Previous research has also found that the *FTO* gene plays a significant role in metabolic diseases and obesity-related diseases [15] (Figure 2). Previous research has also shown that the *Fat Mass Obesity (FTO)* gene is a polygenic risk factor for obesity. Genetic factors contribute to obesity, approximately 30%–70%, and susceptibility to obesity is due to differences between genotypes. The *FTO* gene is one of the genes involved in obesity. The *FTO* gene encodes the enzyme alpha-ketoglutarate-dependent dioxygenase and is widely expressed in all body tissues. This enzyme has several functions: it regulates adipocyte differentiation and thermogenesis, which indirectly contribute to body fat accumulation [24]. Given the important role of genetic factors in the aetiology of obesity and obesity-related diseases, approaches that utilise genetic information are needed to identify individuals or communities at risk of obesity and obesity-related diseases, such as metabolic and cardiovascular diseases [25]. Polymorphisms in the *FTO* gene are strongly associated with diseases such as type 2 diabetes, obesity, and hypertension [25,26]. The *FTO* gene is the gene most closely associated with obesity and cardiometabolic disorders, with polymorphisms largely driven by excess adipose tissue [27].

A study in a Brazilian population found that the *A* allele of the *FTO* gene rs9939609 was possibly associated with blood pressure during pregnancy, with a greater reduction in blood pressure in the traditional diet group compared to the Dietary Approaches to Stop Hypertension (DASH) diet group. However, this *A* allele was not identified as a risk factor for high blood pressure during pregnancy [28]. This study is consistent with findings in the Madurese population, in which the *A* allele was more frequently observed among pregnant women with PE than among normotensives and may be associated with PE; however, these results require confirmation in further research. Considering the importance of the *FTO* gene as a predisposing factor for PE, the characteristics of pregnant women with PE who tend to be obese are studied. The incidence of PE in the Madurese population is quite high, including cardiovascular diseases that occur in the community, such as hypertension and diabetes mellitus, and in pregnant women with PE, these diseases are often found because there is a family history of hypertension and PE.

This aligns with GWAS, which reports that 250 genes are already associated with obesity, and the most significant gene responsible for the obesity phenotype is the *FTO* gene on chromosome 16 [29]. The *FTO* protein affects the central nervous system and energy balance. It was found that the hypothalamus expresses it widely, and it is believed that this effect is mediated by its impact on energy balance, which the hypothalamus controls. Patients with type 2 diabetes mellitus often experience masked (nocturnal) hypertension. The risk of cardiovascular disease is associated with nocturnal hypertension, which is defined as blood pressure being elevated during a clinical examination, but normal blood pressure is observed outside the clinic. *FTO* is potentially associated with increased nocturnal blood pressure in addition to obesity and BMI [30].

The strengths of this study include being the first to examine genetics in the Madurese population and the high incidence of PE. The limitations of this study include the relatively small sample size from a single ethnic group within the Madurese population. Considering the heterogeneous gestational ages among early- and late-onset PE cases, there is a possibility that this sample is not representative of all pregnant women. Therefore, there is a need to sample the entire Madurese population at each hospital. This sample was limited to one hospital, so it is necessary to explore several genes that are potential target factors for PE, as well as to examine various gestational ages and levels of PE severity to account for differences in BMI and metabolic background between groups.

## Conclusion

The results of this study indicate that the *FTO* gene may be associated with PE in the Madurese population. This is the first study conducted in the Madurese population. The results are similar to those in Iran, where the *AA* genotype was associated with a lower risk of PE. However, this study requires further investigation into gestational age at onset, later stages of PE, PE severity, and other populations outside Madura. The *FTO* gene is also associated with increased maternal weight gain or obesity, as seen in the study results, where a greater proportion of pregnant women with PE were obese.
